# Menopause and Plantar Heel Pain: Findings From a Series of Focus Groups Exploring Lived Experience

**DOI:** 10.1002/jfa2.70154

**Published:** 2026-06-01

**Authors:** Matthew Douglas‐Harris, Richard Wilkins, Jayasree Ramaskandhan, Jagjit Mankelow, Helen Branthwaite

**Affiliations:** ^1^ Department of Trauma and Orthopaedics Newcastle Upon Tyne Hospitals NHS Foundation Trust Newcastle Upon Tyne UK; ^2^ NIHR Newcastle Biomedical Research Centre Newcastle Upon Tyne UK; ^3^ Leeds Institute of Rheumatic and Musculoskeletal Medicine University of Leeds Leeds UK; ^4^ School of Health and Life Sciences Teesside University Middlesbrough UK; ^5^ Centre for Biomechanics and Rehabilitation Technology University of Staffordshire Stoke‐on‐Trent UK

**Keywords:** fascia, menopause, musculoskeletal, pain, perimenopause, plantar fasciitis, plantar heel pain

## Abstract

**Background:**

Plantar heel pain (PHP) is a debilitating problem disproportionately affecting women between the age of 40 and 60 at twice the rate of men, coinciding with perimenopause and menopause. Whether a causative relationship exists is uncertain, but their concurrence presents challenges for both patients and healthcare providers.

**Aim:**

To conduct focus groups to understand people's perception of menopause and PHP. The primary objective was to identify whether people with experience of PHP and menopause or their healthcare providers, thought their PHP was related to menopausal status. The secondary objective was to find out if this population thought this was an important area of research and what the barriers may be for further investigation.

**Methods:**

Following National Institute for Health and Care Research (NIHR) guidance on ‘co‐producing a research project’, 17 participants were invited via VOICE GLOBAL public engagement UK platform to take part in focus groups with 5–6 participants in each session. Participants self‐identified as having experienced PHP and were either perimenopausal or postmenopausal. Each group was asked if they felt that there was an association between PHP and menopausal status; if a link between menopause and PHP was made by their healthcare provider; if research in this area was important and what the barriers may be to further investigation in this area.

**Results:**

Of the 17 participants, 76% did not initially perceive that there to be an association between menopause and PHP and reported that it was not mentioned in their healthcare consultations when they sought help. Some felt stigma remained around the menopause, particularly in ethnic minority communities, and that this may be a barrier to further research. Participants unanimously felt that this was an area that needed more investigation, with improved awareness on the part of healthcare providers being particularly important.

**Conclusions:**

Participants felt the relationship between menopause and PHP requires further investigation, with a focus on understanding the level of awareness of association among healthcare professionals and patients. The implementation of any findings needs to consider ethnic and cultural barriers, and those that may be present in a primary care setting.

**Abbreviations**
MSK: Musculoskeletal
PHP: Plantar heel pain
PPIE: Patient and public involvement and engagement

## 1 Background

Plantar heel pain (PHP), also known as plantar fasciitis, is a highly prevalent problem affecting around 9.6% of the UK population [1]. It is characterised by pain in the heel and can have a debilitating effect on quality of life and activities of daily living (ADL) [2]. Several structures in the foot are affected by PHP, particularly the plantar fascia, which plays a key role in maintaining postural stability [3]. During an episode of PHP, the plantar fascia becomes thickened, and although there is often a feeling of *stiffness* from the patient, mechanically the stiffness is reduced, meaning it is less able to provide stability and effectively transfer force generated by the surrounding musculature [4].

**FIGURE 1:**
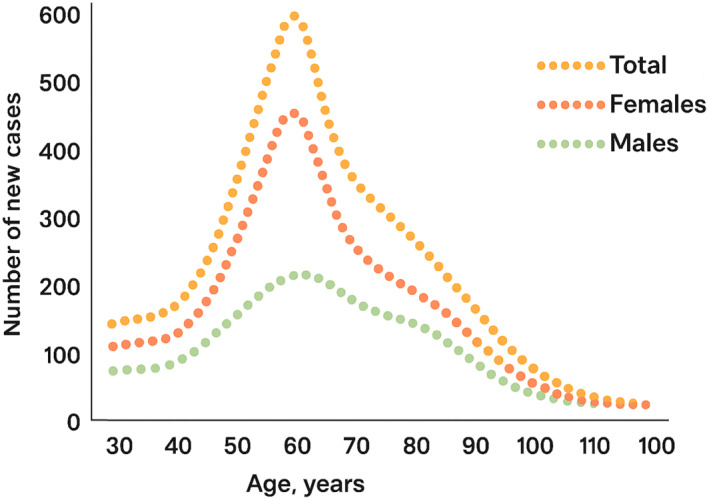
Incidence of plantar heel pain (PHP), separated according to age and gender, adapted from Rasenberg et al. 2019 [5].

The peak incidence of PHP is twice as high in women who are 50 years old than in men of the same age, (11.23–5.54 per 1000, respectively) (Figure 1) [5]. For women, this coincides with the menopause which in the United Kingdom has an average age of 51. The time prior to this (perimenopause) is characterised by a constellation of symptoms across the body, but typically includes hot flushes, sleep disturbances and menstrual irregularity [6].

It has already been established that there are sex differences in the structural and mechanical features of fascial tissue throughout the body, including the plantar fascia, with fascia in females being thinner and stiffer than in men [7, 8, 9]. Fascial tissue itself encompasses the extra‐cellular matrix (ECM), a scaffold responsible for the overall maintenance of fascia. How the ECM interacts with fascial tissue is heavily influenced by hormones in both men and women [10, 11]; however, this variability is particularly notable during the menstrual cycle, where fluctuating hormone levels lead to increased tissue elasticity. This is thought to be a contributing factor to the increased number of knee ligament injuries seen in younger female athletes [12]. However, it is less widely recognised that plantar fascia laxity is also affected by hormone fluctuation during the menstrual cycle, which manifests as reduced postural stability compared to male controls [13].

Menstrual irregularity is also one of the primary features of perimenopause where fluctuating hormone levels may influence musculoskeletal (MSK) function [14]. There are also well understood changes postmenopause, related to reduced muscle mass and bone density, with balance and stability being particularly affected [15]. Muscle and Joint pain have been a feature of menopause screening tools for many years [16] with an association recognised in some areas of clinical practice [17]. However, specific investigation regarding its prevalence and impact has only recently been characterised in this population [18].

Although the prevalence of specific pathological processes and precise pathomechanics is not well understood [19, 20], there is an emerging body of knowledge that indicates lower sex hormones; primarily oestrogen, testosterone and progesterone are associated with higher levels of chronic MSK pain in women [21]. Additionally, individuals with preexisting chronic pain symptoms may experience a higher burden of symptoms during menopause [22].

It is understood that the individual experience of chronic pain can be modulated by factors beyond those of the initial injury or problem. Sleep quality and deprivation [23], the psychological and social context in which the individual is functioning [24] and the perceived stress associated with pain levels [25] as well as attitudes and beliefs about the pain [26] can all modulate the way an individual experiences pain. This is a reciprocal dynamic that can lead to increasing cycles of disability, where the accumulation of these factors can affect long term functional capacity and cognition [27, 28].

These features may all occur to varying degrees during menopause, particularly considering the high prevalence of sleep problems and reduced cognitive function. Therefore, it is important to consider the overlapping presence of musculoskeletal pathology, chronic pain and menopausal symptoms, and how this can lead to a cycle of worsening pain and function [29, 30]. To compound these factors, it is not understood whether patients or healthcare providers perceive there to be an association between menopausal status and PHP or even wider MSK pain; or what the current level of knowledge is for healthcare providers working in this area. Both national and international priority setting work has identified menopause and musculoskeletal health as an area for further research as well as empowering and enabling women to identify earlier when they may be experiencing menopausal symptoms [31, 32].

Therefore, the main aim of this work was to understand the views and perspectives of those who have lived experience of both PHP and menopausal symptoms. The primary objective was to identify in people with experience of PHP and menopause whether they or the healthcare providers they encountered, thought their PHP was related to menopausal status. The secondary objectives were to establish if this population thought PHP and menopause was an area in need of further research, what the barriers may be to this and what needed to be considered in further work. A public partnership approach was used to develop the framework within which the focus groups were carried out [33].

## 2 Methods

Public Partnerships: The NIHR emphasises the need for research to be undertaken in partnership with the public, with the first step being *Research Inclusion* [34] where participants of research projects are involved throughout the process in a *Co‐Production* capacity [33]. Therefore, it was determined that a series of focus groups would be appropriate to meet the objectives and to provide direction on further work. NHS England guidance [35] was referenced in the design and administration of the groups.

Recruitment: Following an advert on the VOICE Global public engagement platform, associated with the National Innovation Centre Ageing [36], in the United Kingdom from February to April 2024, participants were invited to apply to join a series of focus groups. Inclusion criteria were described in the advertisement as those who ‘Have suffered or are suffering with plantar fasciitis and are peri, mid or post‐menopausal’. The term ‘plantar fasciitis’ was used for the advertisement as it is more widely recognised in current healthcare literature, the term ‘plantar heel pain’ has only appeared in the literature more recently and may not have been as widely known.

Participants: 39 individuals between the age of 27 and 84 who self‐identified with the above inclusion criteria applied to take part (32 female, 2 male and 5 who chose not to disclose their gender). Participants had the option to provide a short statement regarding their suitability for inclusion and ethnic background. The statements were reviewed, and 18 participants were invited to attend in line with the principles of purposeful sampling [37]. The broad age range of applicants reflects the impact of PHP and menopause on the population and given the exploratory nature of this work it was important to include a wide range of views and experience. The focus groups took place on Microsoft Teams, with 6 participants in each group. 1 participant could not attend on the day of the focus group so there were 17 female participant responses recorded overall; attendees were remunerated for their time in line with NIHR guidance [35, 38].

Consent: Ethical approval was not sought as this was an initial PPIE exercise; however, principles of Good Clinical Practice were adhered to throughout the process. Participants were advised prior to the focus groups that the session would be recorded and transcribed, and their participation indicated consent for this. Introductions were made at the start of the session, and the participants were advised that a series of questions would be asked about their experiences. Verbal consent was then gained again from each participant for the rest of the focus group session to be recorded, transcribed, analysed and findings disseminated, including direct quotations.

Focus group protocol: Following introductions, each group was asked a range of questions designed to provide structure for the discussion, to generate information on the importance and impact of the topic area and to understand potential barriers to further work (Table 1).

**TABLE 1:** Questions asked to the focus group to instigate discussion.

1: Do you or did you associate heel pain or plantar fasciitis with the menopause?
2: Was the menopause discussed when you sought healthcare advice for your plantar fasciitis?
3: Do you think this is an important area for me to research?
4: What are the barriers in the conversation around menopause and plantar fasciitis?
5: Is there anything else to consider in this area of research?

The transcription of each focus group was analysed, with numerical answers tallied for questions 1–2, whereas questions 3–5 were designed to stimulate broader discussion. The study was conducted within a qualitative theoretical framework, and inductive thematic analysis was used to identify key themes from participant responses according to Braun and Clarke's framework [39]. Although this was not a qualitative study, this approach facilitated a more robust synthesis of the information generated to ensure that key themes of the discussion were captured. This included themes that occurred by frequency of discussion within the same group or independently across the three groups conducted. Reflexive awareness was maintained throughout the focus groups sessions and subsequent analysis, with the lead author (MDH) having no lived experience of menopause. While focus groups participants did not report gender to be a barrier to investigation of this topic, both the research team and participants acknowledged the potential for unconscious bias.

## 3 Results

**FIGURE 2:**
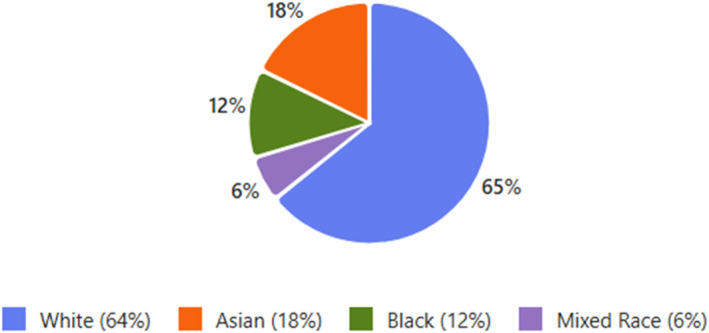
Ethnic demographics of the participants.

Participants were from a variety of ethnic backgrounds with 11 White (64%), 2 Black (12%), 3 Asian (18%) and 1 mixed race (6%) (Figure 2), with a mean age of 52 (27–71). The key finding from the focus groups was that most participants, and the healthcare providers they encountered, did not perceive there to be an association between PHP and menopausal status. Wider focus group discussion was wide ranging and covered several themes in addition to the structured questions asked (Table 2). The primary area of divergent views was regarding the continued stigma concerning menopause.

**TABLE 2:** Overview of results with themes and subthemes identified.

1: Do you or did you associate heel pain or plantar fasciitis with the menopause?
Theme: Initial nonassociation
Subtheme: Most participants did not connect PHP to menopause until prompted.
Theme: Some perceived an association
Subtheme: A minority (or their clinicians) suspected hormone involvement.
Theme: Hormone replacement therapy (HRT) and symptom reflection
Subtheme: Some noticed fewer foot pains on HRT
2: Was the menopause discussed when you sought healthcare advice for your plantar fasciitis?
Theme: Rarely discussed
Subtheme: Menopause not raised by GPs, physiotherapists or podiatrists.
Theme: Knowledge gaps
Subthemes: Most clinicians (often male or those younger) did not make an association
Theme: Exceptions
Subthemes: Some clinicians did consider a hormonal component
3: Do you think this is an important area for me to research?
Theme: Strong support
Subthemes: All groups perceived there to be an evidence gap
Theme: Why it matters
Subthemes: Validation, awareness, targeted treatment, reduced self‐blame and effect on mood and sleep
4: What are the barriers in the conversation around menopause and plantar fasciitis?
Theme: Healthcare barriers
Subthemes: Limited menopause knowledge in MSK pathways, time limits on consultations
Theme: Social and cultural stigma
Subthemes: Menopause taboo, ethnic minority barriers and generational norms
Theme: System capacity and equity
Subthemes: NHS capacity and access compared to private treatments
5: Is there anything else to consider in this area of research?
Theme: Education and training
Subthemes: Focus on GP's, physiotherapists and podiatrists
Theme: Early and inclusive outreach
Subthemes: Menopause awareness in schools, culturally tailored
Theme: Implementation and mobilisation
Subthemes: How will generated evidence be used to improve care?

**Question 1: Do you or did you associate heel pain or plantar fasciitis with the menopause?**

Of the participants, 76% did not initially associate PHP with their menopausal status. One participant suspected their PHP may be related to hormonal changes and sought help from a healthcare provider. Two participants only made an association retrospectively when they discussed symptoms with friends of a similar age. In most cases, the healthcare professional was a general practitioner (GP).

…the second time when I was in my 50s and by that point, other friends had it as well. A few of them had mentioned, is it linked to the menopause and none of us could come up with any sort of theory. Why? So, we did start talking about it.

(65 White)

One participant made more of an association with their increase in weight around this time rather than menopause. Another who was a keen runner associated their PHP with a general running related injury.

**Question 2: Was the menopause discussed when you sought healthcare advice for your plantar fasciitis?**

One participant reported that the healthcare professional they saw considered a link between PHP and menopausal status.

He mentioned that my hormonal changes, generally during menopause can lead to joint pain and stiffness.

(27 White)

Many felt that initial healthcare advice regarding both heel pain and symptoms associated with menopause did not fully meet their needs, necessitating the pursuit of private treatment and alternative therapies.

No, I find in general GPs don't really know much about menopause, let alone making a connection like that.

(49 Asian)

My HRT was not right, so I had to go private for that. So again… I had to do a lot of my own research.

(47 British Asian)

**Question 3: Do you think this is an important area for me to research?**

Overall, participants in all three groups felt that this was an important area to research further and that it would improve clinician and patient awareness. Participants felt that this was important as it would reduce the amount of self‐blame, improve the amount of research in women's health and provide more targeted treatment.

Absolutely. There's not enough research on Women's Health, especially menopause.

(49 Asian)

Absolutely… very important areas.

(50 Black)

The paradox of foot pain being a barrier to physical activity was raised, particularly when this is an essential part of both healthy ageing and chronic pain management. If capacity to do this is diminished, then the negative effects can be wide ranging.

We should all be exercising and if this is a barrier to exercise.

then it'll have a serious impact on people's health.

(52 White)

…you don't want to shuffle around in your 40s and 50s, you really don't. You feel like you're 90, and that's not a good thing.

(65 White)

Some participants also felt strongly that the research was important, but only if it led to changes in treatment or interventions or prevented unnecessary higher risk interventions.

I agree that it's important, but… is there something else that can be done if we find out there is a correlation, because what is the point in finding out there is a correlation if… it won't change anything in the potential approach… to treatment or to the intervention that can be offered.

(59 White)

The importance of awareness before menopause was raised, so that those affected can put strategies in place including improved education and awareness. Particularly where the cumulative effects of menopause may influence the feeling of self and individual agency.

It's an absolute must… so that people don't feel like they're going into the abyss… The fatigue hits you, the brain fog hits you and you do lose a sense of yourself… I felt I was incapacitated.

(47 British Asian)

**Question 4: What are the barriers in the conversation around menopause and plantar fasciitis?**

A significant barrier identified was that of ethnicity and religious background, with a generational trend to not seek medical care.

The other barrier is from an ethnic and cultural and Islamic perspective.

(47 British Asian)

One specific reason for this was that of a language barrier between patient and healthcare provider, which would stop someone seeking medical care in the first instance.

There's a cultural, religious barrier that they won't go to the GP. They won't discuss their problems or there's a language barrier…my mother or grandmother won't go to the doctor and they won't ask. They will suffer and they have suffered and they've kept things.

(47 British Asian)

The most common barrier mentioned was people not linking menopause with foot pathology in the first instance.

The barrier is not recognising the association.

(54 White)

…the general treatment for menopause would not be the treatment for plantar fasciitis. I guess that's a barrier to them being discussed in the context of the two being connected.

(58 White)

This came up separately in all three focus groups. A further barrier was that of medical jargon, making it difficult for patients to engage.

The language and technology can also be a barrier, so you know most times when they use this medical jargon or overly technical terms, it can be alienating and confusing.

(27 White)

All three groups agreed that menopause was more widely acknowledged now and taken more seriously.

The menopause is a big issue for people. It always has been, but I think it's more recognised now in society.

(66 White)

I feel it's only in the last few years that it's come. People are taking it seriously.

(49 Asian)

However, there were also contrasting sentiments from other participants that this was not the case, with comments around persisting misogyny in women's health issues and the feeling that it was still a taboo subject. There was a feeling that some still felt unable to talk about menopause and that this itself was a barrier.

I find it difficult to talk about because there's just a lack of understanding it's almost treated as a joke really… If you don't feel there's understanding, then you're not likely to engage in a conversation about it.

(58 White)

You can't talk to people about it, so it becomes a very huge problem and a very big barrier as well.

(50 Black)

There's this stigma that it is a personal problem or that we've just been dramatic.

(27 White)

I think it still is a bit of a taboo subject… and I don't feel there's enough education around it.

(59 White)

There's just a lot of misogyny in Women's Health issues.

(49 Asian)

The interaction with healthcare professionals and the healthcare system was also discussed as a barrier, with the response of the healthcare professional being particularly influential. One participant also raised that beyond the prescription of HRT, there was little further treatment or advice offered. There was a comment that questioned whether further intervention should be found if there were insufficient resources to effectively implement it.

So that just shut down the conversation. And I think when you're not listening to, and you're not validated, and you don't have the confidence…

(54 British Asian)

I didn't receive a sympathetic or what I felt to be an understanding and listening. GP in that situation.

(61 White)

So, they're like, put you on HRT, but they don't kind of want to sort of engage with anything else really, that's kind of up to you to kind of sort of sort out.

(52 White)

…and with our strapped health care system as it is now. Perhaps we don't want to uncover new treatments and new things that we should be doing. Because there's already sufficient pressure on the health service

(61 White)

**Question 5: Is there anything else to consider in this area of research?**

Further comments were varied, with some tying into the themes of the previous issues raised, including education for healthcare professionals and patients, so that an association may be made in future.

A little bit of education. For the GPs and the community teams, podiatrists, physiotherapists. Or even nursing just to link the two.

(71 White)

The differing experiences of persisting pain was also raised, again with comments on the impact this has on the ability to exercise and maintain good mental health.

I do think you have to be careful that how pain is perceived because it is perceived differently by different people.

(65 White)

You know it stops you exercising… that's going to impact mental health, which is already impacted potentially by the menopause.

(58 White)

A comment was raised regarding knowledge mobilisation and ensuring that any information generated was used effectively by the appropriate clinicians.

What are you planning to do with information… how are you going to make patients life better and different? … what is your next step and who's going to be the target audience? Which healthcare professional do you think will be able… to get involved in that in that process?

(59 White)

Other comments during the focus groups that did not answer the specific questions, but nonetheless provided valuable insight concerned the gender of healthcare provider being important in receiving the right care for other sex‐specific conditions.

…when I was young, I had endometriosis which was a nightmare, and I didn't get proper treatment until I had a female consultant after many years.

(65 White)

…another guy who I saw was quite a young lad. So, I don't think it would have even entered his mind to consider that it was part of the menopause.

(54 White)

However, gender alone was not necessarily an indicator of a satisfactory consultation, with age or experience also being a key factor.

…it was a female GP, and she was she was quite young… She asked my symptoms and she says no, it doesn't look like you're going through that.

(54 British Asian)

## 4 Discussion

The aim of these focus groups was to understand the views of those who had experience of PHP and menopause. The purpose of doing this was to inform the parameters for further research and to understand where research priorities may lie for those affected by PHP and menopause. Participant responses were varied and insightful, covering a broad range of areas including the constraints of the health system, clinician and patient knowledge gaps, socio‐cultural stigma and the challenges facing ethnic minority groups.

### 4.1 The Association of Menopause, PHP and MSK Pain

Considering so few participants associated their PHP symptoms with menopause raises enquiry as to whether MSK pain in other parts of the body may have a stronger association with menopause, and whether this would influence their consultation or help‐seeking behaviour. Conversely, it may be that there is no association between MSK pain and menopause at all. However, it is understood that many women may not seek help at the time of the menopause due to a lack of knowledge about the full spectrum of symptoms that can occur during it as well as the misattribution of symptoms to other causes [40, 41]. This is reflected in the menopause research priorities, where preparing people for menopause, helping them recognise when it is happening and prompting them to seek help, are key research priorities [31].

### 4.2 Variation in Profession Practice

Only one participant encountered a healthcare provider who considered a link between their menopausal status and PHP. This suggests that exploring the perceptions of healthcare providers on this issue is important, to understand if they feel there is a link between menopause and PHP and how this would impact their practice. If they do not associate PHP specifically, then there could be other features that the professional group may associate more strongly with menopausal status, such as vasomotor, sleep or psychological symptoms [42].

A potential disparity is that patients with PHP may present to different professionals depending on their local healthcare provision structure. GP surgeries are often the first point of contact, where patients may be seen by either a GP or a registered nurse within the practice. Alternatively, they may be redirected to local physiotherapy or podiatry services for treatment. In these settings, patients may receive differing treatment depending on the background of the healthcare professional they see, with podiatrists traditionally focusing on footwear and orthoses and physiotherapists emphasising exercise intervention [43].

### 4.3 Barriers to the Conversation

Several barriers were identified by the participants in developing the conversation around menopause and PHP. This included broader cultural and societal barriers as well as those related to the dynamic of the healthcare consultation itself. It is noted that females have a stronger preference for female clinicians in a general primary care setting [44], and it may be assumed that this leads to better consultation satisfaction; however, according to participants' views, a perceived lack of knowledge and the young age of clinicians still resulted in less satisfactory outcomes. However, in specialist menopause clinics, the gender or age of the clinician is of much less importance, with ‘experience,’ ‘knowledge’ and ‘ability’ being much more highly valued [45]. There were divergent views on the social acceptance of discourse regarding menopause, with some participants feeling it was more openly discussed and taken seriously. However, many felt stigma remained and described a persisting taboo, particularly in some communities and ethnic groups.

### 4.4 Ethnic and Religious Barriers

Several participants felt ethnicity was a key barrier in the conversation around menopause as well as women's health more broadly. This echoes the well‐established literature base on disparity in menopause care, race and ethnicity [46, 47, 48]. It is understood that women from Asian and African backgrounds are often less likely to medicalise menopause or seek medical assistance for symptoms [40]. Understanding this dynamic further is another key priority in menopause research, where it is recognised that experiences will vary across countries, cultures and ethnicities [31]. There are frameworks within which practitioners can provide a more responsive level of care for patients depending on their societal and cultural backgrounds [49], however, whether their application is widespread and commonly used in practice is not known.

### 4.5 Implementation of Findings

It was suggested by participants that even if a link between menopause and PHP was clearly identified, implementing changes in practice may be problematic due to restrictions on clinician's time in a primary care setting and the financial implications of new treatments [50]. Therefore, it was suggested that further research should also focus on how clinical practice could be practically changed to implement any new treatment modalities.

This suggests a wide range of stakeholders should be included in developing and directing further research, including patients, health care professionals and commissioning services. This will ensure new knowledge can be effectively translated into clinical practice and healthcare infrastructure. As well as existing patients with direct experience of menopause and MSK pain, it may be beneficial to include premenopausal women without symptoms, to understand potential barriers and beliefs that may influence help seeking behaviours. There are various frameworks for this but having usable and applicable knowledge that informs patient care will be the primary aim [51, 52].

## 5 Impact

Thanks to the participants being willing to share their experiences these focus groups have helped inform the direction of further research. A wide range of topics were raised, each of which could be followed up in depth. As a priority, understanding the views and practices of healthcare professionals was a priority. Therefore, the next stage of work involves a larger scale survey‐based project investigating the knowledge and views of certain professional groups. This would not only involve their experience of treating plantar heel pain but also other foot and ankle pathology. Thanks to the information generated by this group further work will also be better positioned to navigate potential barriers to investigation and implementation. It has emphasised the need for a highly collaborative approach to further investigation that includes a wide range of stakeholders.

Participants of the groups were invited to review the draft manuscript of this publication and 12 participants responded with feedback. This included improved clarity around the key findings and more readable format. Suggestions were made regarding accurate description of participant characteristics and using visuals to illustrate this. Reviewers felt the manuscript was an accurate reflection of the group they attended and that their voice was sufficiently heard. Lastly, participants felt the debilitating impact of PHP needed greater emphasis, particularly where it intersected with menopause. These participants have also consented to be involved in further work as a ‘lived experience advisory group’ to inform the direction of further research in a coproduction capacity.

## 6 Limitations

Information from the focus groups achieved the main objectives. Further work that reaches a wider audience will provide the breadth and depth of information needed to provide a more robust foundation on which patient care can be improved. The use of the VOICE UK patient participation platform means those who do not have English as first language or were not aware of the platform were unable to take part. This method of recruitment also excludes those who are not digitally literate or otherwise engaged in online communities. The selection bias from patients expressing interest in the focus groups and being selected to take part based on purposive sampling, also induces areas of bias that will need to be mitigated in further work, particularly as it may attract those with a negative experience of the subject area.

## 7 Conclusions

These focus groups identified a wide range of issues. Participants felt the link between menopause and plantar heel pain is an area of healthcare that requires further investigation. At the time of onset, most did not perceive there to be an association between PHP and menopausal status and did not feel their healthcare provider made an association either. Participants were supportive of more research to improve the treatment journey for patients with PHP and to raise awareness among healthcare providers and patients of a possible association with menopausal status. These focus groups also identified important barriers to conducting further research, including ethnic and cultural barriers, as well as those affecting healthcare delivery in the health system.

## Author Contributions

**Matthew Douglas‐Harris:** conceptualisation, funding acquisition, data curation, formal analysis, investigation, writing – original draft, writing – review and editing. **Richard Wilkins:** writing – review and editing, supervision. **Jayasree Ramaskandhan:** writing – review and editing. **Jagjit Mankelow:** writing – review and editing. **Helen Branthwaite:** conceptualisation, investigation, methodology, project administration, writing – review and editing, supervision.

## Funding

Time to complete this work was funded by the Newcastle NIHR Biomedical Research Centre. Participant remuneration was funded by a grant from the Community for Allied Health Professionals in Research (CAPHR).

## Ethics Statement

Ethical approval was not sought due to this being a PPIE exercise; however, Good Clinical Practice principles were adhered to throughout the project.

## Consent

All participants consented to be part of the focus groups and for the session to be transcribed and findings published.

## Conflicts of Interest

The authors declare no conflicts of interest.

## Supporting information


Supporting Information S1

## Data Availability

Data sharing is not applicable to this article as no datasets were generated or analysed during the current study.
